# Evaluation of the Functional and Sensory Properties of Probiotic Whey Cheese with Herbs in Vacuum or Modified Atmosphere Packaging

**DOI:** 10.17113/ftb.62.02.24.8421

**Published:** 2024-06

**Authors:** Gülfem Ünal, Didem Ekin Akyıl, Ayşe Sibel Akalın

**Affiliations:** Department of Dairy Technology, Faculty of Agriculture, Ege University, 35100 Bornova, Izmir, Turkey

**Keywords:** oregano, rosemary, lor cheese, probiotic viability, antioxidant activity

## Abstract

**Research background:**

Some herbs provide functional properties to foods, especially their antibacterial and antioxidant properties. On the other hand, modified atmosphere packaging is being considered as an alternative to vacuum packaging to preserve the functional and sensory properties of foods. Since the shelf life of whey cheese is quite short, different packaging methods such as modified atmosphere packaging are favoured. Besides, the addition of herbs both gives flavour to the cheese and improves its functional properties.

**Experimental approach:**

In the present study, oregano (*Origanum onites*) or rosemary (*Rosmarinus officinalis*) was added to probiotic whey cheese (lor) containing *Lactobacillus acidophilus* La-5 and *Bifidobacterium lactis* Bb-12 under modified atmosphere packaging (MAP) (80 % CO_2_ and 20 % N_2_) or vacuum packaging. The physicochemical, microbiological and sensory properties as well as antioxidant and proteolytic activities of the cheese samples were determined.

**Results and conclusions:**

The addition of herbs did not negatively affect the viable counts of *B. lactis* and *L. acidophilus*, and the cheese samples contained at least 8 log CFU/g of both probiotic bacteria for 35 days. MAP improved the viability of *B. lactis* and *L. acidophilus* in cheese with rosemary during the first few weeks of storage compared to vacuum packaging. The addition of herbs significantly increased the total phenolic content and antioxidant activity under both MAP and vacuum. MAP improved the antioxidant activity of lor cheese with added herbs on days 14 and 28 more than vacuum packaging. Lor cheese with rosemary under MAP conditions showed the highest DPPH˙ (2,2,-diphenyl-1-picrylhydrazyl) scavenging activity and also the highest proteolytic activity throughout storage. The sample with rosemary under MAP had the highest taste and aroma scores throughout the entire storage period. Fortification with herb and MAP offers advantages in the production of whey cheese. The use of rosemary and modified atmosphere packaging makes it possible to achieve high viability of probiotic bacteria, total phenolic content, antioxidant activity and sensory acceptance in lor cheese.

**Novelty and scientific contribution:**

This is the first study in which both different herbs and different packaging methods were applied to probiotic whey cheese (lor). The study shows that the functional properties of whey cheese can be improved by using different herbs under different packaging conditions. Among the analysed properties of the product, the improvement of the viability of probiotic bacteria is particularly valuable for human health. Thus, it contributes to the science of functional food and enables the use of these parameters in some other foods.

## INTRODUCTION

Whey is an industrial by-product of cheese obtained by coagulation of milk and elimination of the curd during cheese production. It is a good source of organic substances containing different amounts of lactose, proteins, minerals, fat and vitamins depending on the cheese production processes and the milk used (*1*). Whey is processed into whey cheese in many countries such as Italy (ricotta), Greece (manouri or anthotyro), Norway (brunost), Germany (Ziger), France (brocciu), Spain (requesón) and Turkey (lor). The production of whey cheese is based on the heat treatment of whey, which leads to the denaturation of α-lactalbumin and β–lactoglobulin (*2*). Most of the whey produced in Turkey is used for the production of fresh cheese called lor cheese. The processing method of lor cheese varies depending on the region and consumption habits (*3*).

Functional foods in the category of dairy products or functional dairy products contribute to human health beyond the daily diet and thus promote the well-being of individuals. They contain a wide range of bioactive compounds that can promote health and prevent disease. Probiotics are often used in the production of functional dairy products. Products containing probiotics account for the largest proportion of functional dairy products. Probiotics are defined by the Food and Agriculture Organization of the United Nations (FAO) and the World Health Organization (WHO) as "live microorganisms that provide health benefits to the host when administered in adequate amounts" (*4*). The probiotic market is growing rapidly worldwide, and dairy products today make up the majority of foods containing probiotics. Recent estimates show that the probiotics market will grow at a compound annual growth rate (CAGR) of 8.7 % from its current value of USD 63.11 billion in 2021 to USD 133.92 billion by 2030 (*5*).

Apart from a high nutritional value, cheese can also be a probiotic carrier as it provides an anaerobic medium for probiotic bacteria caused by the protein-fat content. This complex form of cheese supports the survival of probiotic bacteria by protecting them from the acidic environment of the gastrointestinal tract (*6*). In the studies with various whey cheeses containing probiotics, an adequate number of viable bacteria were obtained (*7*-*10*). Cheese was also said to have a positive effect on some diseases. Cheese contains high amounts of saturated fatty acids (SFAs), which are commonly associated with an increase in blood LDL-cholesterol, often considered an important marker for cardiovascular disease (CVD) risk. However, recent studies have shown that other nutrients and the chain length of the SFAs in food have a much greater impact on this relationship. Cheese has been reported to have a positive to neutral effect on CVD risk due to its matrix and vitamin K2 content. Vitamin K2 has been considered a potential nutrient for the inhibition of vascular calcification in some human studies (*11*, *12*).

On the other hand, the composition, water activity, acidity, storage media and type of packaging are known to influence microbial spoilage and thus the shelf life of foods (*2*). Therefore, the production, packaging and storage of fresh cheese should be carefully monitored as it has a short shelf life under aerobic conditions (*13*). In recent years, natural methods have been preferred to protect foods and extend their shelf life. Some herbs are potential alternatives to prevent food spoilage as they have strong antimicrobial activity against foodborne pathogens. In addition, herbs draw attention in food fortification for their flavouring properties and strong antioxidant activity due to their high content of phenolic compounds (*14*).

*Origanum onites* L. (oregano) and *Rosmarinus officinalis* L. (rosemary) are both aromatic plants from the Lamiaceae family that grow in the Mediterranean region. Due to its antiseptic, antimicrobial and antioxidant activities, oregano is used in the food industry, in alcoholic beverages, cooking and in perfumery due to its flavour and odour (*15*). According to Becer *et al.* (*16*), *Origanum onites* L. has a high total phenolic content and the main components of its essential oil are carvacrol, γ-terpinene and *p*-cymene. *Rosmarinus officialis* L. is classified as a woody and aromatic plant and can be added to food as a flavouring ingredient or in a traditional medicine due to its anti-inflammatory, antidiabetic, antimicrobial and antiviral properties. In addition, rosemary has superior antioxidant activity because of its high content of phenolic acids, mainly rosmarinic acid (*17*).

Oregano and rosemary have been used in different studies for their potential antioxidant and antimicrobial effects in cheese production (*14*, *17*-*20*). Modified atmosphere packaging (MAP) has become a preferred packaging method due to increasing consumer demand for fresh and preservative-free food products. In this method, food is packed in an atmosphere-covered material containing different amounts of CO_2_ and N_2_ to reduce the physicochemical changes and oxidation reactions, thus prolonging the shelf life and improving the appearance of the food (*21*-*23*). Some studies have investigated the influence of MAP on some properties of fresh soft cheeses such as mozzarella, queso fresco, Minas frescal and domiati (*23*-*26*).

Fresh whey cheese can predominantly be contaminated after obtaining the curd; packaging therefore seems necessary and useful to limit microbial contamination. Considering that the shelf life of fresh whey cheese is less than 7 days, this method has received considerable attention (*13*). Several studies also investigated the role of the use of MAP on the shelf life of different whey cheeses such as requeijão, anthotryros, Myzithra Kalathaki, ricotta and skuta (*1*, *2*, *13*, *21*, *27*). On the other hand, a few studies investigated the physicochemical, microbiological and sensory properties of lor cheese stored under different packaging conditions including MAP. Temiz *et al.* (*22*) found that the MAP with 70 % CO_2_ and 30 % N_2_ was more effective against food spoilage bacteria, yeast and mould in lor cheese than vacuum packaging and the other MAP variants (40 % CO_2_ and 60 % N_2_, 60 % CO_2_ and 40 % N_2_). The authors also observed that cheese under MAP with 60 and 70 % CO_2_ showed good sensory properties for 45 days, while air and vacuum-packed cheese samples were not acceptable after 10 days of storage. In another study, the influence of MAP1 (80 % CO_2_ and 20 % N_2_) and MAP2 (60 % CO_2_ and 40 % N_2_) compared to air and vacuum packaging on the microbiological and sensory properties of lor cheese was investigated. MAP1 was more useful for inhibiting the growth of yeast and mould, total Enterobacteriaceae and viable count. Lor cheese packed in modified atmosphere showed favourable organoleptic properties for 20 days, while control cheese samples showed very low sensory scores after 10–15 days of storage (*28*).

We also tried different CO_2_ and N_2_ volume fractions for packaging lor cheese and the best results for shelf life were obtained with 80 % CO_2_ and 20 % N_2_ (*29*). Even though the use of MAP to preserve different kinds of whey cheese has been investigated, the effect of fortification with herbs (oregano or rosemary) and probiotic bacteria (*Lactobacillus acidophilus* La-5 and *Bifidobacterium lactis* Bb-12) on the functional properties of whey cheese (lor) during storage remains unknown. The aim of this study is to evaluate the physicochemical, microbiological and sensory properties as well as the antioxidant activities of probiotic lor cheese with oregano or rosemary under vacuum packaging or MAP (80 % CO_2_ and 20 % N_2_).

## MATERIALS AND METHODS

### Whey, starter cultures, herbs, packaging material and gas mixture

Kashar cheese whey with 6.83 % total dry matter, 0.70 % fat, 3.49 % protein and pH=6.29 was obtained from Sakıpaga Dairy Products Company (Menemen, Izmir, Turkey).

The freeze-dried direct vat set (DVS) starter cultures of *Bifidobacterium lactis* Bb-12 and *Lactobacillus acidophilus* La-5 (Chr. Hansen A/S, Hørsholm, Denmark) were used after an activation step (30 min at 37 °C) in ultra-high temperature (UHT)-sterilised skimmed milk (Pinar Dairy Products, Izmir, Turkey).

UV-irradiated oregano (*Origanum onites*) and rosemary (*Rosmarinus officinalis*) were purchased from Omeroglu Agricultural Products Company, Kemalpasa, Izmir, Turkey.

The packaging material was polyethylene/terephthalate/polyamide barrier pouches with a thickness 50–210 μm, an oxygen permeability of ≥3 cm^3^/(m^2^·day·MPa), nitrogen permeability of ≥3.5 cm^3^/(m^2^·day·MPa) and a water vapour permeability of ≥5 g/(m^2^·day) at 100 % relative humidity. The gas mixture (80 % CO_2_ and 20 % N_2_) used in the modified atmosphere packaging for the experimental cheese samples was provided by Linde Group, Izmir, Turkey.

### The production of probiotic lor cheese

The cheese samples were produced in the plant of Sakıpaga Dairy Products (Menemen, Izmir, Turkey). Kashar cheese whey was heated at 90 °C for 5 min and then 0.1 % salt was added. The curd was collected from the surface and drained for 15 min at room temperature before pressing.

The lor cheese was then inoculated with a pre-activated inoculum containing *Lactobacillus acidophilus* and *Bifidobacterium lactis* to reach 10^8^ CFU/g and then stirred before dividing into three groups. The first group was the control cheese, which did not contain any herbs. The other two groups were enriched with 2 % oregano or rosemary. Each group was packed as a 100 g portion under two conditions: modified atmosphere (80 % CO_2_ and 20 % N_2_) and vacuum. The packaging of all experimental cheese samples was completed within 1 h and they were stored at 4 °C for 35 days for the experiments.

Physicochemical analyses (total solids, fat, protein and salt) were carried out at the beginning of storage. Microbiological and sensory properties as well as titratable acidity and pH value were determined weekly during the 35 days of storage. Antioxidant activity, phenolic content and proteolytic activity were measured on days 1, 14 and 28 of storage.

### Physicochemical analyses

The pH value of the cheese was determined using a pH meter (model 211; Hanna Instruments, Woonsocket, RI, USA). The total solids were determined with a standard method of measuring mass loss after drying (*30*). The fat content of the cheese samples was analysed using the Gerber method (*20*). Titratable acidity was expressed as g lactic acid per 100 g after mixing 10 g cheese sample with 10 mL hot distilled water and titrating with 0.1 M NaOH using 1 % phenolphthalein indicator. The salt concentration was determined by the titration with 0.1 M AgNO_3_ (*30*). Total protein was determined using the Kjeldahl method and calculated by multiplying the total nitrogen content by a factor of 6.38 according to AOAC method 920.123-1920 (*31*).

### Microbiological analysis

The viability of probiotic bacteria in the whey cheese samples was determined according to Akalın and Ünal (*32*). A mass of 10 g of each sample was diluted with 90 mL of Ringer’s solution (Ringer tablets; Merck, Darmstadt, Germany) and mixed uniformly using a vortex mixer (Reax top; Heidolph, Wood Dale, IL, USA). Serial dilutions were then prepared and the bacteria were counted using the pour plate technique. MRS NNLP (nalidixic acid, neomycin sulfate, lithium chloride and paramomycin sulfate, Merck) agar was used to determine the viable counts of *Bifidobacterium animalis* ssp. *lactis* Bb-12. The incubation was performed anaerobically (in anaerobic jars; Merck) at 37 °C for 72 h. The viability of *Lactobacillus acidophilus* La-5 was determined on MRS-sorbitol (Merck) agar and after incubation at 37 °C for 48 h in anaerobic jars (Merck).

### Determination of total phenolic content and antioxidant activity

Total phenolic content (TPC) of cheese samples was determined using Folin-Ciocalteu method (*33*). A mass of 10 g of cheese sample was centrifuged (model 3-16KL; Sigma, Shropshire, UK) at 9383×*g* for 25 min, filtered using Whatman no. 1 filter paper (Global Life Sciences Solutions Operations UK Ltd, Little Chalfont, UK) and the supernatant was used for analysis. A volume of 0.1 mL of sample extract was mixed with 6 mL of dH_2_O and 0.5 mL Folin-Ciocalteu reactive reagent (Carlo Erba Reagents, Val de Reuil, France) and left for 2 min. Then, 1.5 mL of *w*(sodium carbonate)=20 % (Merck) were added. After keeping the mixture at room temperature in dark for 2 h the absorbance was read using spectrophotometer (Spekol 1300; Analytik Jena AG, Jena, Germany) at 760 nm. The TPC of cheese samples was compared to a gallic acid standard curve and the total phenolic content was expressed in milligrams of gallic acid (Sigma-Aldrich, Merck, St. Louis, MO, USA) equivalents (GAE) per litre of sample. The gallic acid standard curve with the correlation coefficient R^2^=0.9987 was calculated using the following equation:


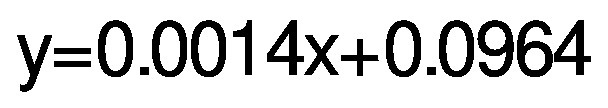
 /1/

The antioxidant activity was determined using 2,2,-diphenyl-1-picrylhydrazyl (DPPH˙; Sigma-Aldrich, Merck) radical scavenging activity method (*34*). A concentration of 0.1 mmol/L DPPH˙ solution was prepared in 95 % ethanol. A volume of 8 mL of this solution was placed in a 50-mL centrifuge tube and mixed with 2 mL of cheese sample or 95 % ethanol (as control), vortexed (Reax top; Heidolph) thoroughly and then incubated at room temperature in the dark for 30 min. The samples were then centrifuged (model 3-16KL; Sigma) at 9383×*g* and room temperature for 10 min. Supernatants were filtered using Whatman no. 40 filter paper (Global Life Sciences Solutions Operations UK Ltd). Absorbance of each sample was measured at 517 nm. Trolox (6-hydroxy-2,5,7,8-tetramethylchroman-2-carboxylic acid, Sigma-Aldrich, Merck) was used as a reference antioxidant at a concentration of 0.25 mg/mL. All spectrophotometric analyses were done at 517 nm using an UV-Vis spectrophotometer (Spekol 1300; Analytik Jena).

### Determination of proteolytic activity

The proteolytic activity of cheese samples was determined using the *o*-phthaldialdehyde (OPA) method, which evaluates the released amino acids and peptides (*35*). In the method, OPA reagent (50 mL of 1000 mmol/L sodium tetraborate, 5 mL of 20 % sodium-dodecyl-sulphate, 80 mg OPA dissolved in 2 mL of methanol and 200 μL of β-mercaptoethanol topped up with dH_2_O to a final volume of 100 mL) was used and the absorbance of the solutions was measured using a spectrophotometer (Spekol 1300; Analytik Jena) at 340 nm. The water extract of cheese was prepared by mixing 5 g of cheese sample with 5 mL of 24 % trichloroacetic acid (Sigma-Aldrich, Merck) and the mixture was left at room temperature for 1 h. After centrifugation of the mixture at 3743×*g* and 4 °C for 20 min, it was filtered through Whatman no. 42 filter paper (Global Life Sciences Solutions Operations UK Ltd) and the supernatant was used for analysis.

### Evaluation of sensory properties

The sensory properties were evaluated according to the International Dairy Federation (IDF) (*36*). Eight experienced academics (4 men, 4 women, aged 30–55) from the Dairy Technology Department (Ege University, Izmir, Turkey), who are experts in the evaluation of the organoleptic methods of cheese, conducted the study. The evaluated sensory properties were appearance, consistency, odour and taste. The scores were based on a 5-point hedonic scale (1=dislike extremely, 5=like extremely). The scoring was done individually and the cheese samples coded with 3 digits were given to the panellists in plastic containers. Water was also given to rinse their mouths.

### Statistical analysis

The experiments, including cheese production, were carried out in triplicate. They were analysed by one-way ANOVA using the general linear model (GLM) of the STATISTICA software, v. 25.0 (*37*). The mean values were compared with the Duncan’s multiple range test at the p<0.05 level.

### RESULTS AND DISCUSSION

### Physicochemical properties

**Fig. 1:**
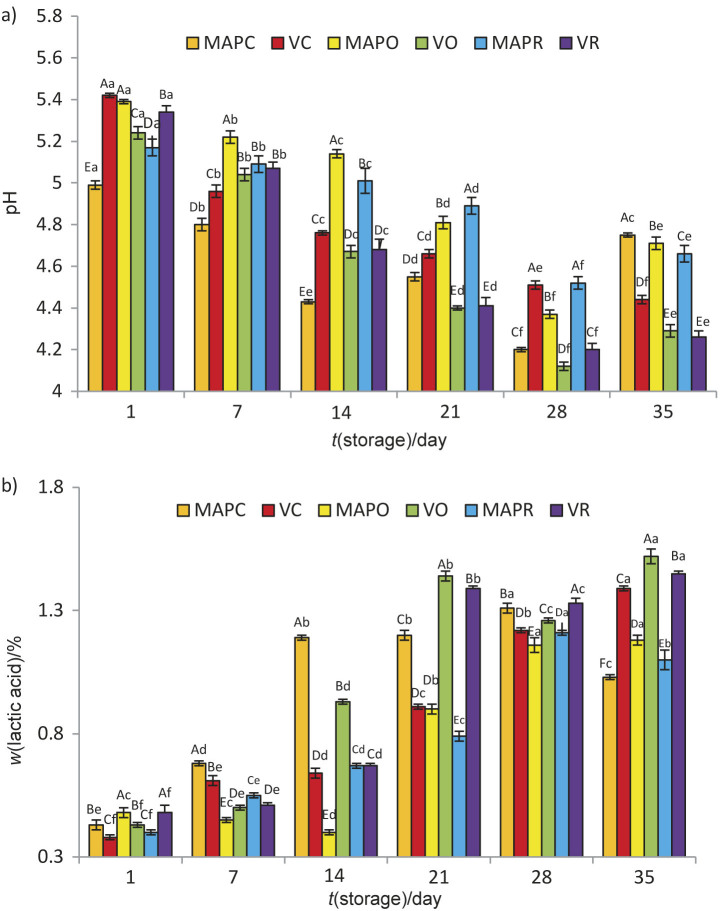
The values of: a) pH and b) titratable acidity expressed as lactic acid of experimental cheese samples during storage. Different capital letters above bars indicate significant differences among samples in the same storage period (p<0.05). Different lower-case letters in superscript indicate significant differences among different storage periods of the same sample (p<0.05). MAPC=control probiotic lor cheese under modified atmosphere packaging (MAP), VC=control probiotic lor cheese under vacuum, MAPO=probiotic lor cheese containing 2 % oregano under MAP, VO=probiotic lor cheese containing 2 % oregano under vacuum, MAPR=probiotic lor cheese containing 2 % rosemary under MAP, VR=probiotic lor cheese containing 2 % rosemary under vacuum

The mass fractions of total solids, protein, fat and salt are given in Table 1. As expected, the supplementation of lor cheese with oregano or rosemary increased its total solids mass fraction (p<0.05), whereas neither the addition of herbs nor the packaging condition significantly affected the fat, protein and salt mass fractions (p>0.05). The results obtained for the protein, total solid and fat mass fraction of control samples stored under vacuum and MAP conditions were consistent with the results of Temiz *et al.* (*22*). The pH and titratable acidity of the samples varied between 4.12–5.42 and 0.38–1.52 %, respectively, during storage (Fig. 1). Although there was a slight fluctuation towards the day 35, the pH decreased in all cheese samples (p<0.05) throughout the storage in parallel with the increase in acidity. The increase in acidity values can be caused by post-acidification, which is related to the degradation of sugars by non-starter lactic acid bacteria and probiotics during storage (*10*). Similar changes in pH values were observed in lor cheese by Temiz *et al.* (*22*), Irkin (*28*) and Akpinar *et al.* (*20*) and in some other whey cheeses in the study by Papaioannou *et al.* (*13*), Cabral *et al.* (*25*) and Silva *et al.* (*10*). Compared to other studies, the lower pH values ​​in our study may be caused by the presence of probiotic bacteria in the cheese samples. Almeida *et al.* (*38*) reported that the culture composition in fresh cheese whey affects the pH and the acidifying rates of the product.

**Table 1:** Composition of probiotic lor cheese

Cheese	*w*/(g/100 g)			
sample	Total solid	Fat	Protein	Salt
MAPC	(31.5±0.5)^BC^	(16.0±1.2)^A^	(10.0±0.1)^A^	(0.33±0.05)^A^
VC	(30.8±0.4)^C^	(16.0±1.2)^A^	(10.1±0.3)^A^	(0.30±0.00)^A^
MAPO	(33.1±0.3)^A^	(16.0±1.2)^A^	(10.0±0.2)^A^	(0.30±0.00)^A^
VO	(33.0±0.7)^A^	(15.00±0.00)^A^	(10.04±0.04)^A^	(0.30±0.00)^A^
MAPR	(32.3±0.9)^AB^	(16.0±1.2)^A^	(10.2±0.3)^A^	(0.30±0.00)^A^
VR	(32.8±1.0)^A^	(15.00±0.00)^A^	(10.00±0.04)^A^	(0.30±0.00)^A^

Mean values±standard deviations in the same column with different uppercase letters in superscript are significantly different (p<0.05). MAPC=control probiotic lor cheese under modified atmosphere packaging (MAP), VC=control probiotic lor cheese under vacuum, MAPO=probiotic lor cheese containing 2 % oregano under MAP, VO=probiotic lor cheese containing 2 % oregano under vacuum, MAPR=probiotic lor cheese containing 2 % rosemary under MAP, VR=probiotic lor cheese containing 2 % rosemary under vacuum

The type of packaging significantly affected the pH and acidity of the cheese. The pH value of the herb-free control cheese packed under MAP conditions was lower than in vacuum packaging (p<0.05). The carbonic acid formed due to the presence of CO_2_ was considered to be the cause of the decrease in pH value under MAP conditions (*21*, *26*). In addition, the observed decrease in pH values may also be caused by the free fatty acids and acidic amino acids produced during lipolysis and proteolysis, respectively (*2*). In some other studies, lower pH values during storage were also observed in packed fresh cheese packed under modified atmosphere than in the samples packed under vacuum (*23*, *24*). In contrast, Silva *et al.* (*10*) could not find significant differences in the pH values ​​of Minas frescal fresh cheese made with probiotic bacteria packed under vacuum or MAP conditions for 21 days.

When lor cheese was analysed under MAP conditions, control cheese generally had higher titratable acidity and lower pH values than the samples with added herbs, especially rosemary, possibly due to its antibacterial effect. de Barros Fernandes *et al.* (*18*) found lower pH values in the control Minas frescal cheese than in the sample with added rosemary essential oil during 15 days of storage. The authors attributed this to the protective effect of rosemary essential oil on the proliferation of spoilage microorganisms. Similarly, Diniz-Silva *et al.* (*39*) observed a higher lactic acid mass fraction and lower pH values in herb-free control probiotic Minas frescal fresh cheese containing *Lactobacillus acidophilus* La-5 than in the sample with oregano and rosemary essential oils in combination. Lower acidity of the probiotic cheese containing essential oils of oregano and rosemary has been attributed to the subinhibitory effect of the essential oils, which slow down the metabolism of *L. acidophilus* La-5 and thus lead to a lower fermentation rate of lactose and a lower concentration of lactic acid.

On the other hand, the acidity did not differ between the lor cheese samples with oregano and rosemary packed under vacuum for 35 days (p>0.05). This may be due to the similar viability of the probiotic bacteria and thus their similar metabolic activity in these cheese samples.

### Microbiological properties

The viable counts of both *Bifidobacterium animalis* ssp. *lactis* Bb-12 and *Lactobacillus acidophilus* La-5 (Table 2) were at least 8 log CFU/g in all experimental cheese samples during refrigerated storage and exceeded the recommended minimum amount (6 log CFU/g) in probiotic foods to ensure a potential benefit (*40*). Both probiotic bacteria were counted between 6 and 8 log CFU/g in cottage cheese (*41*) and more than 8 log CFU/g in prato cheese (*7*). Irkin and Yalcin (*8*) found similar viable counts of *Lactobacillus acidophilus* NRRL B 4495 and *Bifidobacterium animalis* NRRL B41410 of over 7 log CFU/g in salted lor cheese.

**Table 2:** Changes in the viable counts of *Bifidobacterium lactis* and *Lactobacillus acidophilus* during refrigerated storage of probiotic lor cheese

	*t*(storage)/day									
Product	1		7	14		21	28	35		
*N*(*B. lactis*)/(log CFU/g)										
MAPC	(8.7±0.2)^ABa^		(8.7±0.2)^Aa^	(8.7±0.2)^Aa^		(8.7±0.2)^ABa^	(8.7±0.2)^ABa^		(8.8±0.2)^Aa^	
VC	(8.47±0.06)^Ca^		(8.8±0.2)^Aa^	(8.6±0.3)^Aa^		(8.6±0.2)^Ba^	(8.6±0.2)^Ba^		(8.7±0.3)^Aa^	
MAPO	(8.68±0.06)^ABabc^		(8.46±0.05)^Bc^	(8.54±0.03)^ABbc^		(8.48±0.05)^Bbc^	(8.86±0.06)^Aa^		(8.8±0.4)^Aab^	
VO	(8.79±0.02)^Aa^		(8.67±0.02)^Ab^	(8.56±0.05)^ABc^		(8.57±0.05)^Bc^	(8.83±0.06)^Aa^		(8.86±0.03)^Aa^	
MAPR	(8.60±0.06)^BCc^		(8.78±0.01)^Ab^	(8.75±0.07)^Ab^		(8.89±0.02)^Aa^	(8.74±0.02)^ABb^		(8.71±0.06)^Ab^	
VR	(8.07±0.06)^Dd^		(8.19±0.01)^Cc^	(8.26±0.03)^Bb^		(8.91±0.01)^Aa^	(8.91±0.01)^Aa^		(8.86±0.05)^Aa^	
*N*(*L. acidophilus*)/(log CFU/g)										
MAPC	(8.8±0.2)^Aa^	(8.9±0.3)^ABa^		(8.8±0.2)^Ba^	(9.0±0.3)^ABa^		(9.0±0.3)^ABa^			(9.0±0.32)^ABa^
VC	(8.6±0.2)^Ba^	(8.9±0.4)^ABa^		(9.0±0.3)^ABa^	(9.0±0.2)^ABa^		(898±0.3)^Ba^			(9.0±0.3)^ABa^
MAPO	(8.96±0.03)^Aab^	(8.8±0.5)^ABb^		(8.81±0.03)^Bb^	(8.76±0.01)^Bb^		(9.22±0.03)^Aa^			(8.80±0.01)^Bb^
VO	(8.99±0.02)^Ab^	(8.87±0.06)^ABc^		(9.19±0.02)^Aa^	(8.96±0.01)^ABb^		(9.14±0.02)^ABa^			(9.15±0.00)^Aa^
MAPR	(8.97±0.02)^Ae^	(9.06±0.01)^Ac^		(8.99±0.00)^ABd^	(9.17±0.02)^Aa^		(9.13±0.01)^ABb^			(9.06±0.01)^ABc^
VR	(8.16±0.07)^Ce^	(8.53±0.06)^Bd^		(9.01±0.03)^ABc^	(9.16±0.06)^Ab^		(9.15±0.01)^ABb^			(9.29±0.03)^Aa^

Mean values±standard deviations in the same row with different lower-case letters in superscript are significantly different (p<0.05). Mean values±standard deviations in the same column with different capital letters in superscript are significantly different (p<0.05). MAPC=control probiotic lor cheese under modified atmosphere packaging (MAP), VC=control probiotic or cheese under vacuum, MAPO=probiotic lor cheese containing 2 % oregano under MAP, VO=probiotic lor cheese containing 2 % oregano under vacuum, MAPR=probiotic lor cheese containing 2 % rosemary under MAP, VR=probiotic lor cheese containing 2 % rosemary under vacuum

The viable counts of *B. lactis* and *L. acidophilus* did not significantly change in the control cheese and in the cheese with added oregano, while it increased in the cheese with added rosemary during storage (p<0.05). This may be due to the fact that the antimicrobial effect of rosemary initially delayed the growth of probiotic bacteria in cheese. Diniz-Silva *et al.* (*39*) observed also a slowdown of the growth of *L. acidophilus* LA-5 in Minas frescal cheese with oregano and rosemary oils at the beginning of storage.

In addition, packaging condition significantly affected the viable counts of *B. lactis* in cheese samples with rosemary in the first 14 days of storage and in the control cheese on day 1 in favour of MAP. The viability of *B. lactis* in the sample with added oregano did not differ between MAP and vacuum packaging conditions (p>0.05). MAP also resulted in higher counts of *L. acidophilus* in the control sample and the cheese with added rosemary at the beginning and in the first week of storage, respectively, than vacuum packaging (p<0.05). The presence of high amounts of carbon dioxide is a growth factor for anaerobic bacteria (*42*), so the growth of probiotic bacteria may have been positively affected by MAP compared to vacuum packaging, especially in the first days in the samples with added rosemary. These results show that the use of MAP could be a good alternative for packaging in the dairy industry.

Moreover, coliform bacteria and *E. coli* were not detected in the experimental cheese samples during storage (data not shown). This may be due to the increased acidity during storage, the protective effect of the packaging or the antibacterial activity of the herbs used.

### Total phenolic content and antioxidant activity

The total phenolic content (TPC) and DPPH˙ scavenging activity of lor cheese during storage are shown in Table 3. The TPC, expressed as GAE, of lor cheese ranged from 133.6 to 494 mg/L. The addition of herbs significantly increased the TPC of the cheese samples under both MAP and vacuum conditions. The lor cheese with added rosemary had the highest TPC at the beginning of storage, regardless of the type of packaging, while the samples with added oregano or rosemary had the highest TPC on days 14 and 28 under MAP conditions. The storage time significantly influenced the TPC of most lor cheeses. During storage, the TPC was better protected in the herb cheese under MAP conditions than in the vacuum packaging.

**Table 3:** Total phenolic content and antioxidant activity of probiotic lor cheese during storage

*t*(storage)/day			
Product	1	14	28
	TPC as *γ*(GAE)/(mg/L)		
MAPC	(167.6±6.8)^Ca^	(181±20)^Da^	(133.6±2.7)^Eb^
VC	(168.5±9.0)^Ca^	(175.8±14.1)^Da^	(159.0±8.9)^Da^
MAPO	(400±18)^Ba^	(395.4±5.8)^Aa^	(408±16)^Aa^
VO	(396±17)^Ba^	(348.5±9.52)^Cb^	(291.5±7.4)^Cc^
MAPR	(492±9)^Aa^	(409.4±3.2)^Ab^	(409±12)^Ab^
VR	(494±10)^Aa^	(376±17)^Bb^	(348±16)^Bc^
	DPPH˙ scavenging activity/%		
MAPC	(93.65±0.04)^Ca^	(93.8±0.3)^Ca^	(93.8±0.5)^Da^
VC	(93.7±0.3)^Cc^	(94.8±0.3)^Ca^	(94.32±0.06)^Cb^
MAPO	(95.6±1.6)^Ba^	(95.8±0.3)^Aa^	(94.7±1.5)^Aa^
VO	(95.9±0.2)^Ba^	(93.8±0.3)^Bc^	(95.2±0.3)^Bb^
MAPR	(96.9±0.2)^Aa^	(95.83±0.07)^Ab^	(95.670±0.7)^Ab^
VR	(96.7±0.4)^Aa^	(94.8±0.2)^Bc^	(95.5±0.371)^Bb^
Trolox (0.25 mg/mL)	(97.09±0.00)		

Mean values±standard deviations in the same row with different lower-case letters in superscript are significantly different (p<0.05). Mean values±standard deviations in the same column with different capital letters in superscript are significantly different (p<0.05). TPC=total phenolic content, MAPC=control probiotic lor cheese under modified atmosphere packaging (MAP), VC=control probiotic lor cheese under vacuum, MAPO=probiotic lor cheese containing 2 % oregano under MAP, VO=probiotic lor cheese containing 2 % oregano under vacuum, MAPR=probiotic lor cheese containing 2 % rosemary under MAP, VR=probiotic lor cheese containing 2 % rosemary under vacuum

The antioxidant activity of the lor cheese samples was between 93.65 and 96.9 %. The reference antioxidant Trolox (0.25 mg/mL) showed a DPPH˙ scavenging activity of 97.09 %. The control lor cheese had a considerably high DPPH˙ scavenging activity during storage, which is probably due to the antioxidant activity of the whey proteins and the proteolytic activity of the probiotics, which release antioxidant peptides. Proteolytic microorganisms in cheese can produce bioactive peptides with antioxidant activity (*43*).

The addition of oregano or rosemary significantly increased the antioxidant activity (p<0.05), parallel to the TPC results. MAP improved the antioxidant activity more than vacuum packaging of the cheese with added herbs after the first day. The lor cheese with added rosemary under MAP had the highest DPPH˙ scavenging activity throughout storage and similar antioxidant activities were observed in the cheese with added oregano on days 14 and 28 under MAP. The antioxidant activity of the lor cheese samples with both herbs decreased (p<0.05) on day 28 compared to day 1, except for the sample with oregano under MAP conditions (MAPO). The addition of oregano or rosemary has been shown to increase the TPC and DPPH˙ scavenging activity in different cheese samples compared to the plain samples (*14*, *17*, *19*). However, no study has been found on the effects of packaging type on the antioxidant properties of cheese with added herbs. Therefore, this study can be an option for the dairy industry to obtain whey cheese with functional properties.

It is known that the antioxidant activity of oregano and rosemary is due to the phenolic compound content, which is mainly affected by genetic factors, subspecies, cultivation area, harvest time and processing methods (*44*).

The main phenolic compounds in oregano are: carvacrol, thymol, γ-terpinene and *p*-cymene (*15*, *16*). Rosemary also contains phenolic acids (mainly caffeic and rosmarinic acid) and consequently has high antioxidant capacity (*45*).

### Proteolytic activity

**Fig. 2:**
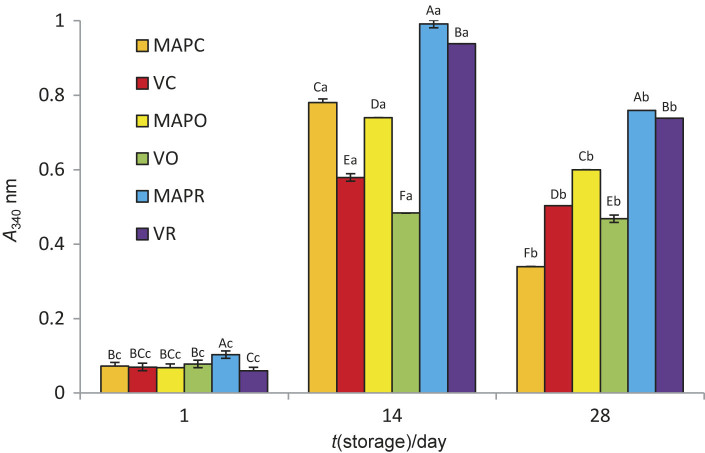
Proteolysis of experimental cheese samples (error bars represent S.D.). Different capital letters above bars indicate significant differences among samples in the same storage period (p<0.05). Different lower-case letters above bars indicate significant differences among different storage periods of the same sample (p<0.05). MAPC=control probiotic lor cheese under modified atmosphere packaging (MAP), VC=control probiotic or cheese under vacuum, MAPO=probiotic lor cheese containing 2 % oregano under MAP, VO=probiotic lor cheese containing 2 % oregano under vacuum, MAPR=probiotic lor cheese containing 2 % rosemary under MAP, VR=probiotic lor cheese containing 2 % rosemary under vacuum

The proteolytic activity of lor cheese is shown in Fig. 2. The proteolytic activity of the experimental cheese was between 0.059-0.991 and fluctuated during storage. It increased in all experimental cheeses on day 14, but decreased on day 28 (p<0.05). The decrease in free NH_3_ on day 28 may be due to the use of free amino groups by the starter culture bacteria.

Proteolysis was more pronounced in the modified atmosphere-packed samples with added herbs than in the vacuum-packed samples. Similarly, probiotic Minas frescal cheese containing *Bifidobacterium animalis* ssp. *lactis* Bb-12 packed under modified atmosphere (50 % CO_2_ and 50 % N_2_) was found to have higher proteolytic activity than the sample packed under vacuum. The absorbance, which indicates proteolytic activity, was lower in Minas frescal cheese than in our cheese samples, which can be attributed to the different manufacturing processes (*10*).

Lor cheese with added rosemary and packed under MAP conditions had the highest proteolytic activity during storage, parallel with the antioxidant activity of the sample (p<0.05). It is known that one of the biological activities formed by bioactive peptides during proteolysis is the antioxidant activity resulting from the hydrolysis of serum proteins (*46*, *47*).

The high proteolysis of the cheese with added rosemary packed in MAP (MAPR) may also be due to the proteolytic activity of the probiotic starter bacteria. It is known that several *Bifidobacterium* strains have low proteolytic activity, while several *L. acidophilus* strains can secrete a large number of peptidases, amino-, di- and tripeptidases and also proline-specific peptidases (*48*). The proteolysis pathways of semi-hard probiotic cheeses made with either a single culture of *L. acidophilus* or a mixed probiotic culture (*Bifidobacterium lactis*, *Lactobacillus paracasei* and *Lactobacillus acidophilus*) were highly analogous and *L. acidophilus* was reported to play a major role in proteolysis due to its stronger proteolytic activity than other culture bacteria. In the same study, the amount of most free amino acids was increased by *L. acidophilus* (*49*). This is consistent with the fact that *L. acidophilus* was more dominant than *B. lactis* in terms of viable bacteria in the MAPR sample in our study.

### Sensory properties

**Fig. 3:**
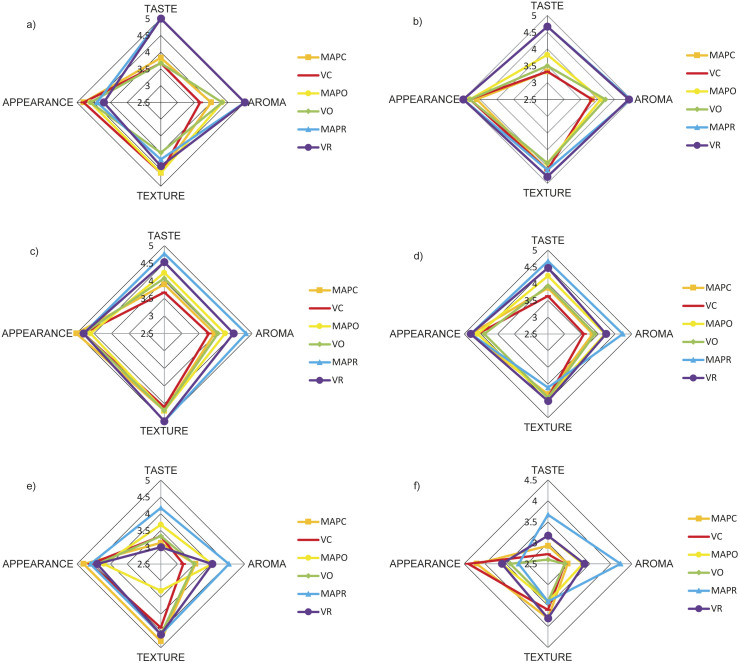
Sensory properties of experimental cheese samples on *t*/day: a) 1, b) 7, c) 14, d) 21, e) 28 and f) 35. MAPC=control probiotic lor cheese under modified atmosphere packaging (MAP), VC=control probiotic lor cheese under vacuum, MAPO=probiotic lor cheese containing 2 % oregano under MAP, VO=probiotic lor cheese containing 2 % oregano under vacuum, MAPR=probiotic lor cheese containing 2 % rosemary under MAP, VR=probiotic lor cheese containing 2 % rosemary under vacuum

The results of sensory evaluation are shown in Fig. 3. The appearance and texture scores of all samples were close to each other during storage (p>0.05). The fresh Minas frescal cheese with and without oregano and rosemary essential oils also received similar scores for texture, colour and appearance during 21 days (*39*). The type of packaging did not affect significantly the taste of the control cheese and the cheese with added oregano added, which was similar to the study by Silva *et al*. (*10*). Maniar *et al.* (*50*) also found that the sensory properties of cheese were not affected by CO_2_. MAP only improved the taste scores of cheese with added rosemary.

The panellists gave the cheese with added rosemary, which was packed in MAP, the highest taste and aroma scores throughout the storage period. Considering that taste and aroma play a very important role in consumer acceptance of a food product, this result indicates that rosemary can be used in the production of cheese and thus contribute to the functional cheese market. The addition of oregano positively affected taste properties of lor cheese on days 14 and 21 compared to the control sample under both packaging conditions. When comparing the aroma of all experimental cheeses, it can be seen that they follow the following order: MAPR>VR>MAPO>VO>MAPC>VC (Fig. 3). Similar tendency in favour of MAP was observed for lor cheese (*22*), domiati cheese (*26*), myzithra kalathaki cheese (*2*) and mozzarella cheese (*24*).

In another study, an oregano oil-in-water nanoemulsion with added Tween 80 did not have any effect on the aromatic profile of anthotyros whey cheese compared to the control cheese without oregano (*14*). In contrast, Akpinar *et al.* (*20*) observed lower sensory records in the lor cheese with added rosemary than in the cheese with thyme, probably due to the different production process and packaging method, unlike in our study.

Significant decrease in taste and aroma properties of all experimental cheeses was observed on day 35 (p<0.05), which was comparable to the study by Irkin (*28*). This could be due to increased proteolysis and thus higher amounts of free NH_3_ in all cheese samples at the end of storage. It has been reported that the increased total free amino acid content released as a result of proteolysis can cause undesirable flavorus such as maltiness and bitterness in cheese (*51*).

Ribeiro *et al.* (*52*) reported that emerging sensory methodologies (ESM) have been used as alternatives to conventional organoleptic analysis in recent years. The authors categorised these methods into four groups: predefined terms, free description, global differences and similarities, and comparisons with references. Studies on cheese using these methods allowed to distinguish the cheese samples according to different sensory parameters (creamy, salty, bitter taste, shiny, spreadable, dull, *etc*.) as well as the type of product and production process (maturation time, culture used, protected designation of origin, milk type, cheese composition, *etc*.). Another innovative method called ’text highlighting technique’, in which consumers are asked to mark passages that they like or dislike in a text with relevant content, has been used for Minas frescal cheese (*53*). If one of these methods can be selected based on the product characteristics, it can be concluded that the dairy industry would benefit from future studies that take them into consideration.

## CONCLUSIONS

Modified atmosphere packaging (MAP) is attracting attention as a means of preserving the functional properties of food. In this study, the influence of the addition of oregano or rosemary on the functional properties of probiotic whey cheese lor was investigated under MAP or vacuum packaging. A high viability of *Bifidobacterium lactis* and *Lactobacillus acidophilus* was observed in all cheese samples in the experiment throughout storage. Total phenolic content and antioxidant activity increased with the addition of herbs and MAP improved the antioxidant activity of lor cheese with added herbs more than vacuum packaging.

Cheese with rosemary addition under MAP generally showed better properties in terms of probiotic viability, DPPH˙ scavenging activity and proteolytic activity than the sample with oregano addition under the same conditions. The cheese with added rosemary packed under modified atmosphere also received better scores for taste and aroma than the cheese with added oregano. As the results show, MAP and the addition of 2 % rosemary offer the advantage of obtaining a functional lor cheese with high antioxidant activity while maintaining acceptable sensory properties and probiotic viability.
